# Supplementary material for: Osmotic response during kidney perfusion with cryoprotectant in isotonic or hypotonic vehicle solution
Source: PeerJ. 2023 Nov 21;11:e16323. doi: 10.7717/peerj.16323 (PMC10668850; doi:10.7717/peerj.16323)
Supplement: Supplemental Information 1 [file peerj-11-16323-s001.pdf]

Supplementary Material

Osmotic Response During Kidney Perfusion with Cryoprotectant in Isotonic or Hypotonic Vehicle Solution

Ross M. Warner[1], Jun Yang[1], Andrew Drake[1], Youngjoo Lee[1], Sarah Nemanic[2], Dave Scott[3], and Adam Z. Higgins[1]

[1] School of Chemical, Biological and Environmental Engineering, Oregon State University, Corvallis, Oregon, USA.
[2] Veterinary Radiology Consulting, LLC, Lebanon, Oregon, USA.
[3] Department of Abdominal Transplantation, Oregon Health & Science University, Portland, Oregon, USA.

Corresponding Author:

Adam Higgins
School of Chemical, Biological and Environmental Engineering
Oregon State University
116 Johnson Hall, 105 SW 26th St
Corvallis, OR 97331-2702, USA
Email address: adam.higgins@oregonstate.edu

Effect of Me_2SO concentration on X-ray attenuation

CT images were acquired of 24-well plates containing varying concentrations of Me_2SO in either water, hypotonic vehicle solution, or isotonic vehicle solution. These images were then analyzed to relate the average grayscale value in the images of each well to the Me_2SO concentration in that well. Figure S1 shows the results of these experiments. As shown in Figure S1A, the background grayscale value in the absence of Me_2SO was different for the different solutions tested. Isotonic vehicle solution had the highest grayscale value, indicating higher X-ray attenuation. Me_2SO increased the grayscale value to a similar extent in each solution. Thus, the results were consolidated by subtracting off the background, resulting in the linear trend shown in Figure S1B.

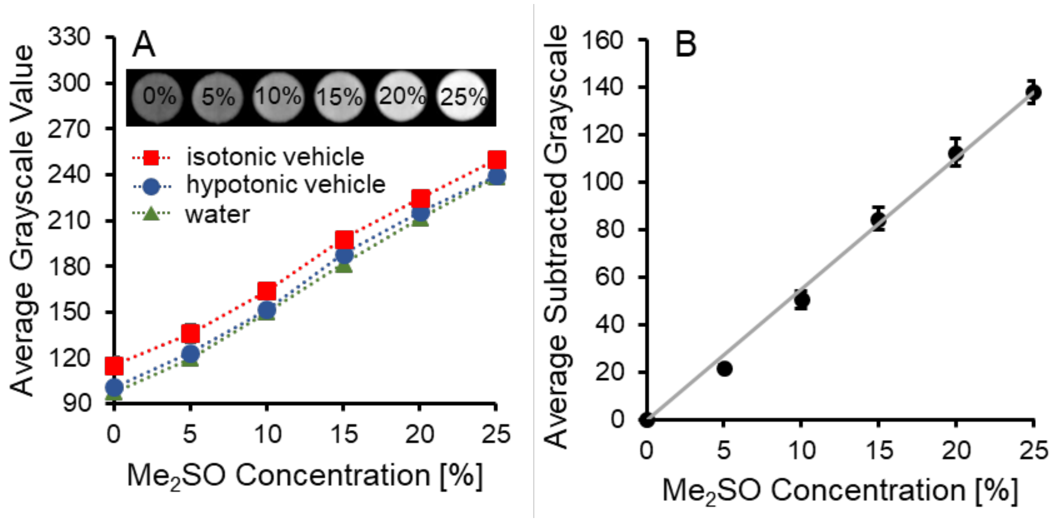

Figure S1. Standard curves between Me_2SO concentration and the grayscale value in CT images. **(A)** Grayscale values for Me_2SO in isotonic vehicle solution, hypotonic vehicle solution, or water. Each solution was tested in triplicate. A representative image is included, showing wells containing Me_2SO in water at concentrations from 0% to 25% m/v. **(B)** Combined measurements for Me_2SO in all three solutions, obtained by subtracting off the background attenuation in the absence of Me_2SO. The best-fit line is shown. Error bars represent the standard deviation.

To estimate the Me_2SO concentration in the kidneys, we used Equation 1, which allows calculation of the Me_2SO concentration using the slope of the calibration curve (Figure S1) and the solids volume fraction x_s. The estimates for the Me_2SO concentration are sensitive to the assumed value of x_s. Table S1 summarizes values of the kidney solids mass fraction reported in the literature.

Table S1. Mass fraction of solids in kidneys from various species.

Solids mass fraction	Species	Kidney region	Reference
13-19%	Pig	Whole kidney	[1]
29%	Human	Whole kidney	[2]
12-19%	Dog	Papilla	[3]
12-16%	Dog	Medulla	[3]
19-21%	Dog	Cortex	[3]
23%	Rat	Whole kidney	[4]
35%	Rat	Whole kidney	[5]
19%	Rat	Whole kidney	[6]

We used a solids volume fraction x_s = 30% to estimate the Me_2SO concentrations reported in the paper. If we assume that the solids mass fraction is approximately equal to the solids volume fraction, the value we used in this paper (x_s = 30%) is on the high end of values reported in the literature. Figure S2 compares Me_2SO concentration estimates using two different assumed values

for the solids volume fraction: $x_s = 0.30$ and $x_s = 0.15$. The assumed value of x_s does not affect the qualitative trends, but it does affect the magnitude of the Me_2SO concentration estimates. Higher concentrations are predicted when the assumed value of x_s is increased. Thus, a limitation of our method is that it relies on an accurate estimate of the solids volume fraction – uncertainty about the value of x_s causes uncertainty in the Me_2SO concentration estimates.

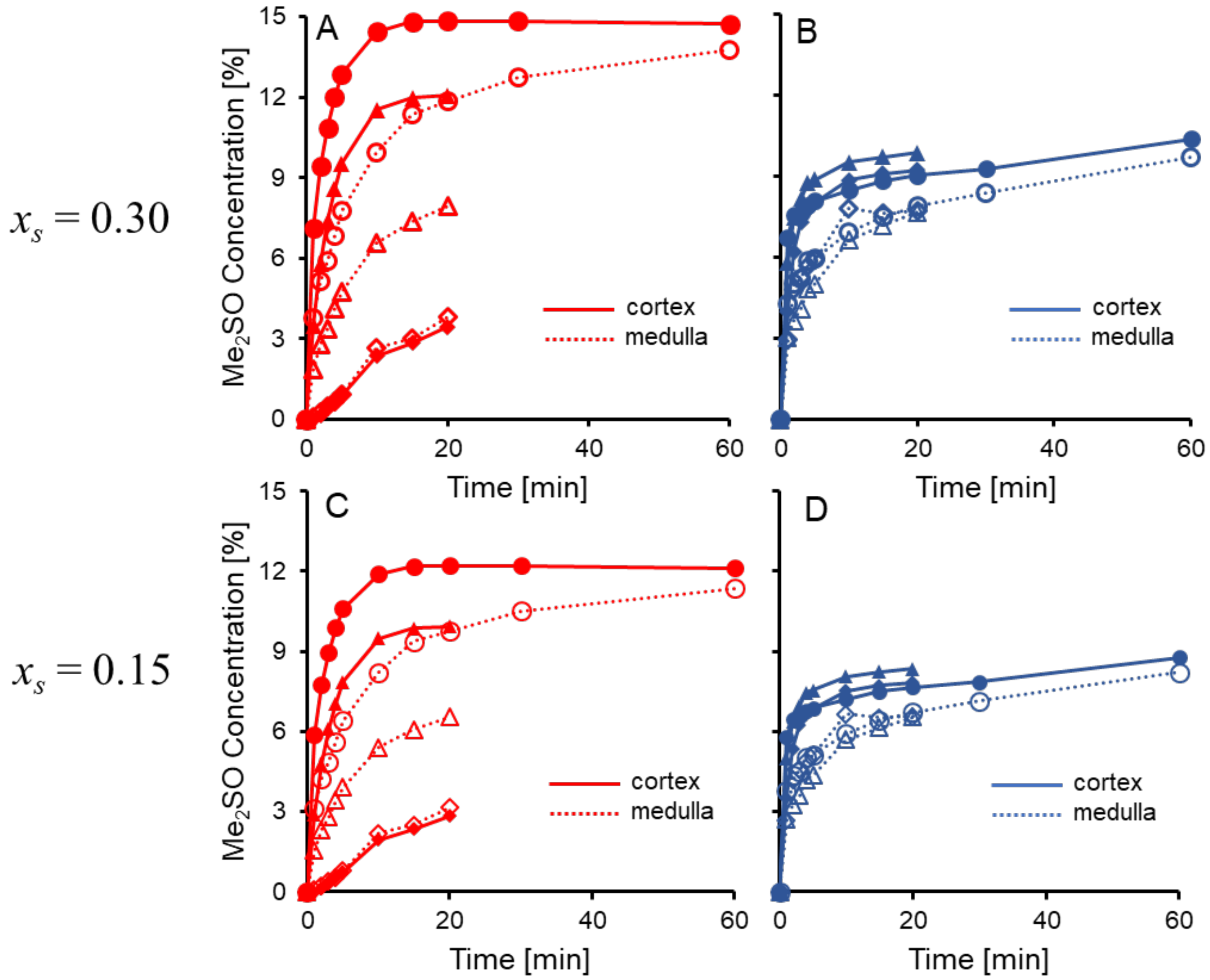

Figure S2. Effect of the assumed value of the kidney solids volume fraction on Me_2SO concentration estimates. Porcine kidneys were perfused with 15% m/v Me_2SO in either isotonic (A, C) or hypotonic (B, D) vehicle solution.

Analysis of CT images

In one case, the orientation of the kidney in the CT scanner did not yield clear images in the coronal plane. In this case, images slices in an oblique plane were analyzed. Cortical and medullary regions were identified as illustrated in Figure S3.

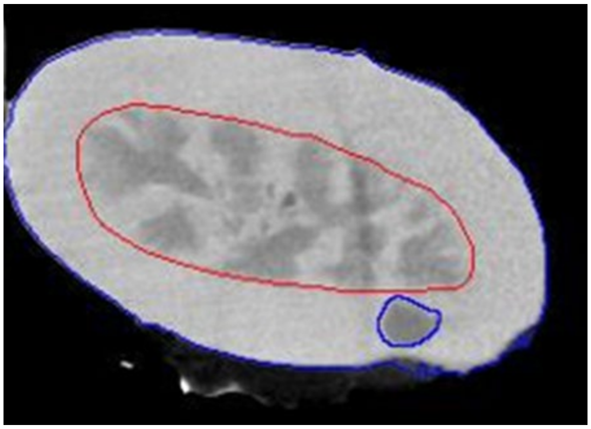

Figure S3. Representative image showing segmentation of a kidney in an oblique plane. Within the red medullary border, it can be seen that there is perhaps some pelvis within the plane at the very center of the image, but this region was deemed insignificant to the analysis. The darker region in the blue cortical border was identified as an abnormality and was segmented out according to the smaller blue border.

Effect of Cold Ischemia Time on Cell Death

To examine the effects of cold ischemia on cell death, we compared the LDH released during the 30 min isotonic vehicle solution equilibration period of all available experiments. The results are presented in Figure S4. LDH release increases substantially as cold ischemia time increases.

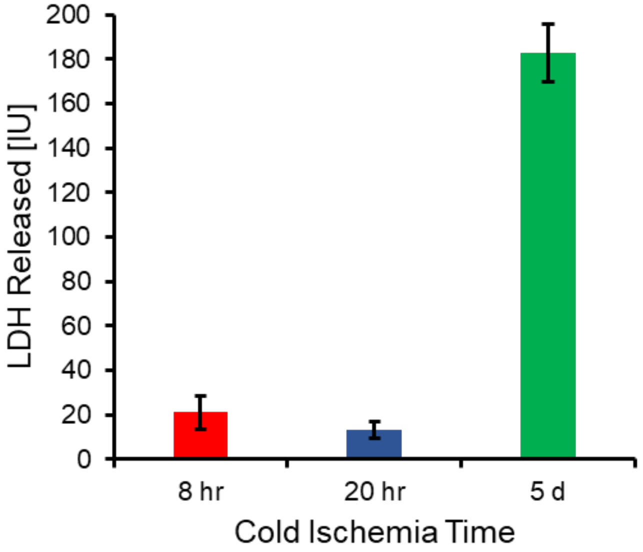

Figure S4. LDH released as a function of cold ischemia time. All kidneys have a warm ischemia time of 25-40 min. The cold ischemia time groups are less than or equal to 8 hours (n = 10), approximately 20 hours (n = 4), and approximately 5 days (n = 6).

Effect of CPA perfusion on vascular resistance

Figure S5 shows vascular resistance values for the kidney group with the lowest warm ischemia time. The vascular resistance was approximately constant after perfusion with CPA in isotonic vehicle solution, but the vascular resistance increased 10-fold within 10 minutes after perfusion with CPA in hypotonic vehicle solution.

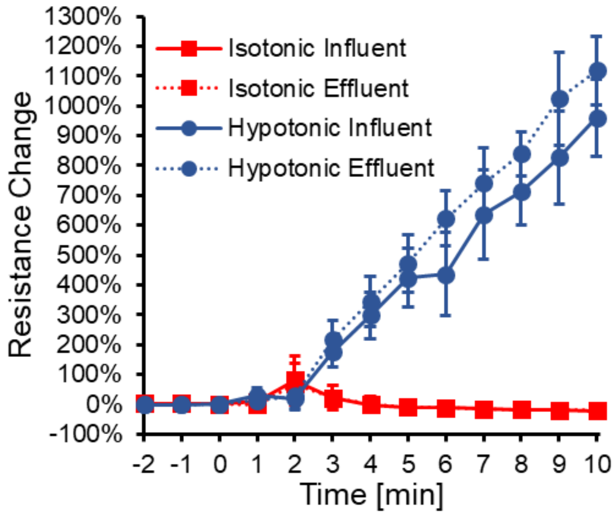

Figure S5. Normalized vascular resistance for porcine kidneys perfused with 10% m/v ethylene glycol in either isotonic (red squares, n = 3) or hypotonic vehicle solution (blue circles, n = 3). The kidneys had an approximate warm ischemia time of 20 min and a cold ischemia time of 3-5 hr. Resistance was calculated as in Pegg et al [7], using either the influent (dashed lines) or

effluent (solid lines) flowrates (see Results for details). At $t = 0$ that average vascular resistance was 3324 ± 503 mm^{-3}.

Pitfalls of Slaughterhouse Kidneys

To obtain kidneys from the slaughterhouse, it was necessary to use methods that only minimally disrupted the slaughterhouse workflow. Kidneys were typically removed by slaughterhouse personnel individually, rather than en bloc, and in some cases, this resulted in kidneys with only a short segment of the renal artery remaining. This made cannulation difficult, and may have resulted in insertion of the cannula past the first arterial bifurcation in some cases (Figure S6, left). The lack of control over the kidney resection process also made it difficult to prevent introduction of air bubbles into the kidney vasculature (Figure S6, right).

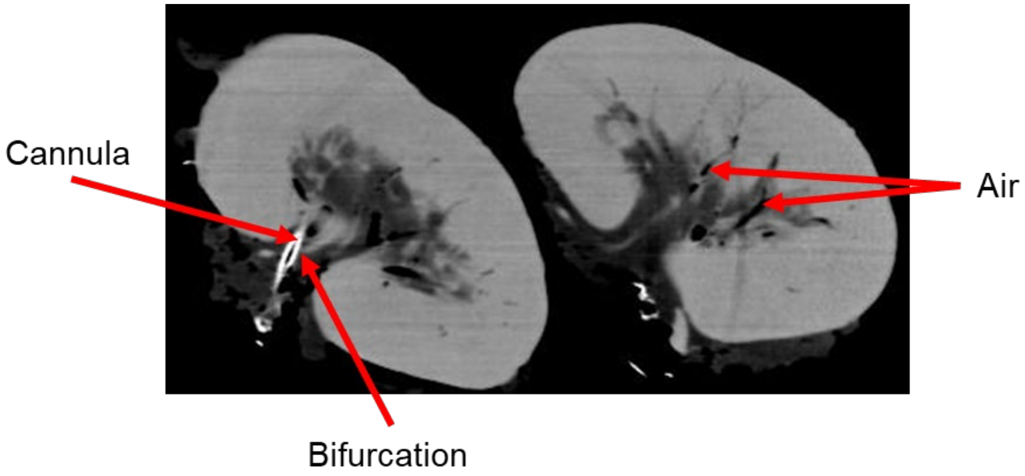

Figure S6. CT image of two kidneys before a perfusion took place. The left kidney shows a cannula position that appears to be past the first arterial bifurcation. The right kidney shows some pockets of air trapped in the vasculature.

References

1. Blum, M.F., et al., *Comparison of normothermic and hypothermic perfusion in porcine kidneys donated after cardiac death.* J Surg Res, 2017. **216**: p. 35-45.
2. Forbes, R.M., A.R. Cooper, and H.H. Mitchell, *The composition of the adult human body as determined by chemical analysis.* J Biol Chem, 1953. **203**(1): p. 359-66.
3. Levitin, H., et al., *Composition of the renal medulla during water diuresis.* J Clin Invest, 1962. **41**: p. 1145-51.
4. Reinoso, R.F., B.A. Telfer, and M. Rowland, *Tissue water content in rats measured by desiccation.* J Pharmacol Toxicol Methods, 1997. **38**(2): p. 87-92.
5. Wang, X.D., et al., *Antioxidant and calcium channel blockers counteract endothelial barrier injury induced by acute pancreatitis in rats.* Scand J Gastroenterol, 1995. **30**(11): p. 1129-36.

6. Lee, K., et al., *Measuring water contents in animal organ tissues using terahertz spectroscopic imaging.* Biomed Opt Express, 2018. **9**(4): p. 1582-1589.
7. Pegg, D.E., et al., *Analysis of the Introduction and Removal of Glycerol in Rabbit Kidneys Using a Krogh Cylinder Model.* Cryobiology, 1986. **23**(2): p. 150-160.
